# 

**Published:** 1911-02-25

**Authors:** 


					Every year the Navy and the Army are discharging
fully-qualified male nurses of exemplary character. The
cost of training these men is necessarily heavy, and until
the Army and Navy Male Nurses' Co-operation was
founded these highly efficient men drifted into unsuitable
channels, where their skilled training found no market
value. It is not generally realised that the TMavy and the
Army are the only general training schools which certify
fully-trained male nurses. The selection of the nurses for
enrolment on the Register of the Co-operation is under
the strictest supervision of a committee formed of officers
on the active list and civilians in the medical profession.
As Reservists, who may be called upon to serve their
country again, they are rendered even more efficient by
varied experience gained in civil nursing. The offices of
the Co-operation are at 47b Welbeck Street, London, W.
MEDICAL APPOINTMENTS, ETC.
Dr. J. E. MacKraine has been elected assistant physi-
cian at the Royal Victoria Hospital, Belfast, in the room
?of the late Dr. M'Quithy.
Dr. Lister has resigned the post of medical officer to
the Morningfield Hospital, Aberdeen.
Mr. P. McEwan, M.A., M.B., Ch.B., F.R.C.S., Edin.,
has been appointed Honorary Assistant Surgeon at the
Bradford Royal Infirmary, to fill the vacancy caused by
the promotion of Mr. James Phillips, F.R.C.S. Edin.
Obituary.
We have to announce the death at Eastbourne, after a
long illness, of James Pitcairn-Bcokless, M.D. Edin., in
his sixty-sixth year.
On January 26, at the " Old Rectory," Sutton-at-Hone,
Kent, Ebenezer David Silver, M.D. Aberdeen 1848, I.M.S.,
?aged ninety-one years.
The death is announced at Liverpool of Dr. Joseph
Dawson Crawford, formerly Senior Surgeon to the Liver-
pool Hospital for Cancer and Skin Diseases, in his seventy-
fifth year.
Mr. Frederick Melland, who had retired from prac-
tice, has died at Manchester in his ninety-third year. He
studied at Sheffield, Manchester, and Paris, qualifying as
L.S.A. in 1840 and M.R.C.S. Eng. in 1841. He then
became assistant physician at the Manchester Royal
Infirmary, and a few years later was appointed one of the
medical officers for the Chorlton Union, only retiring in
1896 upon superannuation. He wTas at that time the
oldest official in the service of the Poor Law authorities.
His zeal for playgrounds and open spaces, says the
Times, was always great, and only a few years ago he
took part in an agitation for the acquisition of a new
park. He retired from practice about ten years ago. He
was actively associated with the Liberal Party, and regu-
larly attended political meetings until he was ninety years
of age. Mr. Melland was twice married. His first wife
was a daughter of Mr. Kelsal, of Rochdale, by whom he
had three children, the eldest daughter of whom married
Mr. Asquith in 1877.
Gifts and Bequests.
The executors of the late Miss E. A. Brown have, in
their discretion, allocated from the residue of her estate
a sum of ?210 to the Royal National Orthopredic Hospital.
Mr. Wm. Evans has generously given a sum of ?50 and
Mr. Wm. Buchler ?26 5s. to the funds of the Leicester In-
firmary. Mr. and Mrs. F. B. Lott have also contributed
?26 5s".
The Coventry and Warwickshire Hospital has received
from the trustees of John Feeney a legacy of ?1,000. This
amount was left to the hospital on the death of the testator
in 1905, but was not to be paid by the executors until a
convenient time, five years after his death. The hospital
has also received ?5 from Mrs. Mary Osborne, in return
for kindness shown to her family.
The sum of ?1,255 realised by the sale of Queen Alex-
andra's facsimile letter has been distributed by her Majesty
to the following institutions : ?500 to " Queen Alexandra
Court," Wimbledon; ?500 to the British Home and Hos-
pital for Incurables at Streatham ; ?129 to the Surgical
Aid Society; ?126 to the Royal National Sanatorium for
Consumption, Bournemouth.
The late Mrs. Sleath, of Oakham, has bequeathed a
legacy of ?10 to the Leicester Infirmary. The Institution
has also received a further payment of ?37 10s. on account
of the legacy bequeathed by the late Mr. T. H. Downing.
The late Mrs. R. C. Christie, of Ribsden, Windlesham,
Surrey, has left ?5,000 to the Zenana Bible and Medical
Mission, to build a new women's hospital in India. Mrs.
Christie has for many years been a liberal supporter of the
Society and has followed its' medical and educational work
with keen interest. The Committee are most thankful for
the substantial help to their work, which will thus be given,
and welcome the prospect of adding a new hospital where
the need is so great. The annual expenditure which will
be involved in its upkeep will be a further call upon the
funds of the Society, which are already inadequate to meet
the growth of the work and to cope with the numerous
opportunities which are opening before Christian workers
in India.
Jottings.
Mrs. Girvin will be at Home to workers of the League
of Mercy, on Saturday, February 25, at 3.30 p.m., at
Ashbourne, Laurie Park Gardens, Sydenham, to meet the
Earl and Countess of Dartmouth.
The Quarterly General and Special Court of the
Governors of the London Hospital, Whitechapel, E., will
be held on Wednesday, March 1, at 3.30 p.m., to receive
the report of the House Committee for the past three
months. This Court is made Special for the purpose of
making and altering certain standing orders relative to the
Medical College, and electing a physician in charge of the
Electrical and Physical treatment department.
The Annual General Meeting of Governors and Sub-
scribers of the Hospital for Women will be held at the
Hospital on Tuesday, March 7 at 3.30 p.m., for the transac-
tion of the following business : To receive the Annual Re
port of the Committee of Management and the Audited
Accounts for the year 1910, to elect Governors of the Hos-
pital, to elect Dr. Richard T. Smith a Consulting Physician
of the Hospital, to elect members of the Committee of
Management, and to elect auditors for the ensuing year.
On conclusion of the ordinary business of the Annual
General Meeting, the meeting will be declared " Special",
for the consideration of certain alterations and additions to
the bye-laws of the Hospital, as approved and recom-
mended by the Committee of Management.
The Countess of Lonsdale has been re-elected Presi-
dent of the Oakham Cottage Hospital, and the following
committees were appointed : Management : Mr. B. A.
Adam, Mr. H. R. Finch, Mr. J. Story, the Right Hon.
G. J. Noel, Mr. H. Sleath, and Mr. E. Worrall. Ladies"
Committee : Mrs. Baird, Mrs. Bradshaw, Mrs. Hodge,.
Mrs. Morris, Mrs. John Royce, Mrs. Tryon, with Mrs.
Furley as hon. secretary. Mr. D. N. Royce was elect
hon. treasurer, and Mr. J. W. Scott auditor.
February 25, 1911 THE HOSPITAL
TYPICAL BARGAINS
at The Times Book Club's
GREAT SALE OF
1 ?3BB?
MEDICAL WORKS
25/-
net. 9/6-
Children in Health and Disease. Vlrltted\ S4LG
A Study of Child Life. By D. Forsyth. ?0/6 l>RICE-
Demy Svo, xix + 362 pp. ... ... ... net
Diseases of the Heart and Arterial
System. Designed to be a practical
presentation of the subject for the use of
students and practitioners of medicine. By
Robert H. Babcock. With 3 Coloured
Plates and 139 Illustrations. Royal Svo,
xxi + 853 pp
Electro Hajmostasis in Operative
Surgery. By Alexander J. C. Skene.
Being a work supplementary to his
" Treatise on the Diseases of Women."
With Eighty Illustrations. Demy 8.0,
!73 PP
Ex Cathedra Essays on Insanity.
By T. Claye Shaw. Crown 8vo, vi + 250
PP
The Food Factor in Disease.
By Franci-i Hare. 2 vols. Demy Svo,
xiv + 497 + viii + 535 pp
Handbook of Electricity in
Medicine. By W. H. Guilleminot.
Translated by W. Deane Butcher. With
8 Plate-f in Colours and 79 Illustrations.
Large post Svo, xxxii + 588 pp. ...
10/6
net.
21-
5/-
net. : 1/9
30/-
net. 15/-
17/-
net. 7/6
SALE
PRICii-
5 h
2/6
3/6
Human Embryology and Morph = jp?"J**c<f
ology. By Arthur Keith. Second
Edition, revised and enlarged. Illustrated J
with 316 figures in the text, of which 64
are new in this edition. Demy Svo, xi + j 12/6
402 pp j net-
A Manual of Diseases of the Nose
and Throat. By Cornelius Godfrey
Coakley. Illustrated with 92 Engravings
and 2 Coloured Plates. Post Svo, 566 pp. j 12/-
Medical Philosophy. Man's
Peculiarities,Weaknesses, Diseases,
Degeneration, and Remedies. By j 7/6
W. Russell. Demy Svo, xiv+504 pp. ... net.
Modern Surgery, General and
Operative, By John Chalmers Da
Costa. Fourth Edition, rewritten and [ i
enlarged, with 707 Illustrations. Royal j 21/- 1
Svo, 1099 pp. ... ... ... ... net. 9/-
The Physiology and Therapeutics
of the Harrogate Waters, Baths
and Climate applied to the Treat-
mentof Chronic Disease. By William
Bain and Wilfrid Edgecumbe. Demy 8vo, j 7/6
xii + 300 pp.  j net. ; 3[~
A System of Surgical Nursing.
With an Appendix containing useful |
Formulae, Emergency Drill, etc. By A. | 9/-
N. M'Gregor. Demy Svo, xi + 554 pp. ... net. 3/6
Intending Purchasers should
PAY AN EARLY VISIT
as no Catalogue has been prepared, and the Stock is strictly limited.
Delivery Free in London and District.
Books to the value of 20/- Carriage Paid to any Railway Station in the Kingdom.
tlbe Tames Booh Club
376 to 384 OXFORD STREET, LONDON, W.
THE LARGEST BOOKSHOP IN THE WORLD.
Telephone: Gerrard 5390 (5 lines). Telegrams: "Unieme, London.'
BUILDINGS CONTEMPLATED AND
IN PROGRESS.
Stoke-on-Trent.?Messrs. Young and Hall are the archi-
tects of the proposed structural alterations to the North
Staffs Infirmary, and not, as stated in our last week's
issue, Messrs. Scrivener and Sons, who are the architects
of the new laundry only, which has recently been erected-
Camelon.?Plans have now been approved for an impor-
tant addition to the Stirling County Council's Fever
Hospital; the extension will provide accommodation for
ten beds in five wards, at an estimated cost of ?1,600.
A feature of the design is that the division walls between
wards are to be of plate glass to assist efficient supervision
with a small staff.
Chichester.?Comprehensive extensions to the Chiches-
ter Infirmary costing close 011 ?14,000 are anticipatedone
of the leading features is a thoroughtly up-to-date operat-
ing theatre. Mr. Ball is the architect.
Egham.?Messrs. King and Son, builders, have just com-
pleted the new Isolation Hospital establishment to serve
Egham and Old Windsor parishes; Mr. Walter Gray of
Egham acted as architect.
Liverpool.?Messrs. Briggs, Wolstenholme and
Thornely, architects, submitted to the select vestry on
February 7 estimates and outlines of a scheme for the pro-
posed epileptic colony at Maghull; it .shows accommodation
for 300 patients at an approximate total cost of ?46,000;
b was decided to submit the scheme to the Local Govern-
ment Board for approval.
Pontypridd.?Under the superintendence of Messrs.
Evans, Williams and Evans, architects, a new cottage hos-
pital has been erected at Pontypridd. Provision is made for
sixteen beds, ten for men, three for women, one separa-
tion ward and two children's cots. The buildings have
been paid for, the contract sum being ?3,590, and the
treasurer has a balance in hand of over ?30 to carry
forward to the furnishing and equipment costs.
THE WEEK'S NOVELTIES.
THE AUSTRAL WINDOW, particulars of which-are
given in our advertisement columns, is the acme of sim-
plicity in window construction. As such it, is. well worth
the attention of hospital architects and constructors,
while its advantages for dwelling houses are very marked.
It must be seen to be appreciated as it deserves, for its sim-
plicity and strength are admirable. It secures perfect ven-
tilation and can be easily cleaned from the inside. A point
in its favour is that the Austral system can be adapted to
existing sashes of the old-fashioned type. The sole licen-
sees in Great Britain are the Austral Window Balance Co.,
Ltd., 3 Macdonald Lane, Corporation Street, Manchester,
from whom illustrated pamphlets on the system may be
obtained.
OZONAIR, Ltd., of 96 Victoria Street, Westminster, S.W?,.
supply special apparatus for pure air generating plant to
institutions. Ozonair in the hall, sick or lecture room,
must be breathed to be fully appreciated, and since its
cost is comparatively low, the question of providing an
institution with the special plant deserves consideration
from hospital architects and committees.
One of the adulterants of cocoa-powder is husk grindings,
and the question of the purity of a cocoa containing a cer-
tain amount of husk was recently the subject of a legal
discussion. CADBURY'S BOURNVILLE COCOA is
absolutely free from such admixture and may be relied
upon as a standard cocoa of the English type which for
delicacy of flavour stands unrivalled. Its food 'Value is
high, and it is pre-eminently one of the cocoas most deserv-
ing the attention of the practitioner and the institutional
worker.
In our advertisement columns appear particulars of the
book bargains to be secured this and next week at the
Times Book Club's SALE OF MEDICAL WORKS. The
prices charged for many standard net books are very much
reduced. No catalogue has been prepared and the stock
should be seen as .soon as possible by intending purchasers.
JflEDICAL APPOINTMENTS.
Full particulars of these vacancies can be obtained on'
reference to our advertisement columns :?
Garloch Mental Hospital.?Resident Physician's Assist-
ant.
Birmingham and Midland Homceopathic Hospital.?-
Resident House Surgeon.
Manchester Children's Hospital.?Resident Medical
Officer.
Tower Hamlets^Dispensary.?Resident Medical Officer.
West Bromwich District Hospital.?Assistant Resident
House Surgeon.
Wolverhampton and Staffordshire General Hospital.?
Resident Surgical Officer.
During 1910 the Newton Abbot Hospital lost by death
three of its prominent supporters on the board of manage-
ment, namely, of Mr. J. Wright, Mr. W. J. Watts,
and Mr. L. Owen Tucker. Mr. Wright was a member of
the committee for twenty years, and during that period
ho was always anxious to do all in his power for the
advancement of the good work of the hospital. Mr. Watts
was the treasurer for the last six years, and Mr. Tucker
had acted as auditor for two years. Both these gentlemen
were greatly interested in the hospital, and anxious to
help in every way. (Mr. Gerald Fox has been elected
auditor in Mr. Tucker'c place.)
GENERAL PRACTITIONERS'
CONTRIBUTIONS.
Tiie Hospital devotes a special page to General
Practitioners' contributions. We therefore invite
from practitioners contributions based upon their
experience in the management of cases, and in the
treatment and diagnosis of disease; especially shall
we be prepared to welcome articles dealing, practi-
cally, with treatment, and with the use and value
of new remedies and methods.
No article should exceed 1,100 word's, and, if
accepted, one guinea for a contribution of this
length will be paid to the writer after publication.
Each communication should be accompanisd by a
stamped directed envelope for the return of the MS.
if found unsuitable.
Contributions should be written, or preferably
typed, on one side of the paper only, and all articles
sent in are accepted upon the distinct understanding
that they are forwarded to The Hospital only.
Suggestions Invited.
The Editor will welcome suggestions for the estab-
lishment of any new section in The Hospital, and
will be glad to supply information on any subject of
interest or importance to members of the profession
in any part of the world.
February 25, 1911 THE HOSPITAL
The Scientific Press Publications
INSTITUTIONAL WORKS.
HOSPITALS AND ASYLUMS OF THE WORLD,
By SIR HENRY BURDETT, K.C.B., K.C.Y.O.
In Four Volumes, with Separate Portfolio containing several hundred Plans.
Vols. I. and II. (ASYLUMS) ... Price ?6 15s. I Vols. III. and IV. (HOSPITALS)
and the Portfolio of Plans ... Price ?9
The Portfolio of Plans (20 in. x 14 in., drawn to uniform scale), Price ?4 14 6
The Complete Work   ?12 12s.
This magnificent work forms a Complete Survey of the Origin, History, Construction, Administration and Management
?of the Hospitals and Asylums of the World, with detailed information concerning the principal Institutions.
Only a few copies remain unsold.
LEDGERS, ACCOUNT BOOKS, STOCK BOOKS of all kinds for large
and small Institutions.
These are supplied-already ruled and printed, or in accordance with the special requirements of institution managers.
ANNUAL REPORTS AND PROSPECTUSES.
The Scientific Pbess are prepared to undertake the production of the Annual Reports and Prospectuses of every
class of Institution, and to supply printed or typewritten copies of testimonials for the officials. Professional Cards.
THE SCIENTIFIC PRESS, LTD.,
28 and 29 Southampton Street, Strand, London, W.C.
THE PROGRESSIVE HOSPITAL
SECRETARY.
ANALYSIS LEDGER.
Specially Ruled In accordance with, and adapted to the
requirements of, the New Index of Classification recently
adopted by the King Edward's Hospital Fund for
London.
"The Ledger is bound in half-basil, green cloth sides, printed
And ruled in best style on good paper, and is in every
way a serviceable book for Hospital use.
Price ?1 12s. 6d.
Carriage extra.
THE SCIENTIFIC PRESS, LTD.,
28 & 29 Southampton St., Strand, London, W.O.

				

